# Biosynthetic polyphosphate enhances osteogenesis of human periodontal ligament stem cells and promotes periodontal bone regeneration in a murine periodontal bone defect model

**DOI:** 10.3389/fbioe.2025.1672295

**Published:** 2025-09-17

**Authors:** Jiaqi Chen, Dongying Lei, Xinyi Liu, Zipeng Chen, Jiaying Li, Liang Huang, Huifen Liu, Xuebin Yang, Wei Wei, Sijing Xie

**Affiliations:** ^1^ Nanjing Stomatological Hospital, Affiliated Hospital of Medical School, Research Institute of Stomatology, Nanjing University, Nanjing, China; ^2^ Central Laboratory of Stomatology, Nanjing Stomatological Hospital, Medical School of Nanjing University, Nanjing, Jiangsu, China; ^3^ Chemistry and Biomedicine Innovation Center (ChemBIC), State Key Laboratory of Coordination Chemistry, School of Chemistry and Chemical Engineering, Nanjing University, Nanjing, China; ^4^ Department of Oral Biology, School of Dentistry, University of Leeds, Leeds, United Kingdom; ^5^ State Key Laboratory of Pharmaceutical Biotechnology, School of Life Sciences, Nanjing University, Nanjing, China

**Keywords:** polyphosphates, hPDLSCs, osteogenic differentiation, periodontal bone regeneration, tissue regeneration

## Abstract

**Introduction:**

Periodontal bone regeneration remains a significant challenge in clinical dentistry due to the complex structure of periodontal tissues and their limited intrinsic regenerative capacity. Innovative biomaterial-based strategies are therefore required. Polyphosphates (Poly(P)) have shown promising regenerative potential; however, conventional chemical synthesis methods are limited by high costs and product impurity concerns.

**Methods:**

We established an eco-friendly biosynthetic strategy using a genetically engineered environmental bacterium overexpressing polyphosphate kinase (PPK1) to produce high-purity polyphosphates (Bio-Poly P) from wastewater-derived phosphate sources. Structural characterization was performed to confirm physicochemical properties. The effects of Bio-Poly P on human periodontal ligament stem cells (hPDLSCs) were assessed by CCK8 assays, qRT-PCR, alkaline phosphatase (ALP) activity, and Alizarin Red staining. *In vivo* osteogenic potential was evaluated using a murine periodontal bone defect model with micro-CT analysis after 4 weeks of implantation.

**Results:**

*In vitro*, Bio-Poly P at 1.25 and 2.5 mg/ml did not reduce hPDLSC proliferation at 24, 48, and 72 h, whereas higher concentrations (≥5 mg/ml) significantly inhibited proliferation (P < 0.0001). At day 7, Bio-Poly P at 0.25, 1.25, and 2.5 mg/ml significantly upregulated *COL1A1* expression (P < 0.0001), while only 1.25 mg/ml enhanced OCN (P < 0.0001) and OPN (P < 0.01). No effect was observed on RUNX2 at this time point. By day 14, all three concentrations significantly increased the expression of *RUNX2, OCN, OPN*, and *COL1A1*. Enhanced ALP activity and calcium deposition were confirmed by biochemical assays and Alizarin Red staining, with the 1.25 mg/ml group showing the greatest mineralization. *In vivo*, Bio-Poly P significantly improved bone mineral density, bone volume/tissue volume ratio, and trabecular thickness compared with untreated defects, with regenerative outcomes comparable to the clinical control Bio-Oss® (P > 0.05).

**Discussion:**

This study demonstrates that Bio-Poly P possesses favorable biosafety and osteoinductive properties, effectively enhancing osteogenic differentiation of hPDLSCs *in vitro* and promoting periodontal bone regeneration *in vivo*. By leveraging a cost-effective and sustainable biosynthetic production method, Bio-Poly P represents a promising alternative to chemically synthesized polyphosphates for clinical periodontal regeneration.

## 1 Introduction

Inorganic polyphosphates (Poly(P)) are linear polymers of orthophosphate that are widely present in mammalian nuclei, mitochondria, lysosomes, and plasma membranes (Kumble and Kornberg, 1995). They play critical roles in mineral metabolism, particularly in bone formation, by regulating calcification and decalcification (Fleisch et al., 1966) and promoting osteoblast proliferation through modulation of mineralization (Leyhausen et al., 1998). Poly(P) has also been reported to influence the expression of osteogenic genes (Tsutsumi et al., 2000) and inhibit dentin resorption in co-culture models of osteoblasts and macrophages (Hacchou et al., 2007). Additionally, Poly(P) stabilizes fibroblast growth factor (FGF), a key modulator of bone matrix mineralization (Kawazoe et al., 2008; Kawazoe et al., 2004). Beyond its role in general bone metabolism, Poly(P) has garnered attention in periodontal research (Gawri et al., 2016). Studies have demonstrated that topical application of Poly(P) can induce connective tissue remodeling and improve gingival inflammation in rat models of periodontitis (Kasuyama et al., 2012). Furthermore, Poly(P) has been reported to stimulate alveolar bone regeneration, suggesting its potential in periodontal tissue engineering (Gawri et al., 2016; Yamaoka et al., 2008). These findings highlight the therapeutic potential of Poly(P) in enhancing osteogenic activity and promoting periodontal tissue regeneration.

**SCHEME 1:**
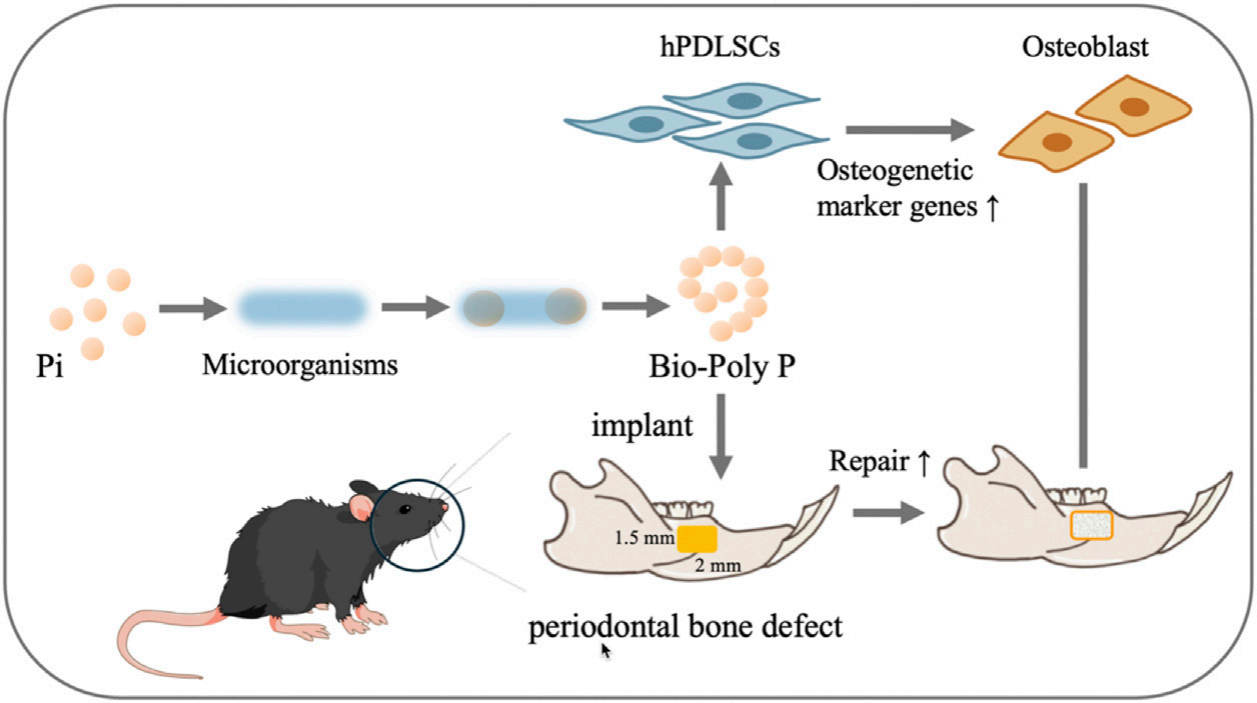
Animal and hPDLSCs experiment procedures. Design and application of Bio-Poly P for treating periodontal bone defects in murine and in hPDLSCs (Pi is the abbreviation of phosphate).

Although Poly(P) holds significant biological relevance, its clinical application remains constrained by the challenges associated with conventional chemical synthesis methods, which involve high-temperature dehydration condensation reactions. This approach faces several challenges, including the need for stringent reaction conditions that influence the average chain length of the resulting product and the production of environmentally harmful waste. These limitations hinder its suitability for biomedical applications.

Recent advancements have developed a biosynthetic approach for Poly(P) production utilizing genetically engineered microorganisms. Wang et al. (2018) reported a novel strategy involving the overexpression of polyphosphate kinase (PPK1) in bacteria via a medium-copy plasmid, enabling the efficient intracellular accumulation of Poly(P) (Wang et al., 2018). Compared to chemical synthesis, this biological method offers several advantages, including milder reaction conditions, reduced environmental impact, and the utilization of sustainable phosphorus sources, such as wastewater. Despite these benefits, the biological effects of biosynthetically produced Poly(P) (Bio-Poly P) on periodontal regeneration have yet to be fully elucidated.

In this study, Bio-Poly P was synthesized via genetically engineered bacteria, unitizing a medium-copy plasmid system. We investigated the effects of Bio-Poly P on the osteogenic differentiation of human periodontal ligament stem cells (hPDLSCs) and its potential to enhance periodontal bone regeneration in a murine model, aiming to establish a foundational understanding for its clinical application in periodontal therapy. Our experimental strategy is presented in Scheme 1.

## 2 Materials and methods

### 2.1 Synthesis and characterization of Bio-Poly P

Bio-Poly P used in this study was generously provided by Professor Wei’s research group. It was biosynthesized using an engineered bacterial model overexpressing polyphosphate kinase (PPK) via a medium-copy plasmid, cultured in nutrient-deficient synthetic wastewater, as previously described (Wang et al., 2018).

The physical appearance of Bio-Poly P was captured using a Canon digital camera (Canon, Japan). Fourier-transform infrared spectroscopy (FTIR; BRUKER TENSOR 27) was used to detect the characteristic phosphate bonds (O–P–O and P=O). For FTIR analysis, 5 mg of the sample was ground into a fine powder and analyzed using the attenuated total reflectance (ATR) method. Powder X-ray diffraction (PXRD; BRUKER D8 ADVANCE) was conducted to assess the crystalline structure of both Bio-Poly P and chemically synthesized polyphosphate. Approximately 20 mg of each sample was ground and placed on a silicon wafer for analysis. Then, the samples were mounted on stubs with conductive adhesive, sputter-coated with gold to enhance conductivity; the surface morphology of the samples was analyzed under scanning electron microscopy (SEM; JEOL SEM-100) with various magnifications.

### 2.2 Chain length measurement of Bio-Poly P

The chain length of Bio-Poly P was determined by 31-P Nuclear Magnetic Resonance (^31^P-NMR). Briefly, a 30 mg sample was dissolved in 400 μL D_2_O and tested in a 400 M liquid NMR system. Urea-PAGE revealed the chain length distribution of Bio-Poly P. A gel solution containing 15% Acr-Bis (29:1) and 7 M urea was polymerized with 30% APS and TEMED, forming a 1 mm thick gel. Samples were mixed with an equal volume of 2 × TBE loading buffer before running. Pre-running was performed for each gel under 200 V for 30 min, then the samples were added and run under 150 V for 45 min. Poly P bands were visualized by DAPI staining.

### 2.3 Preparation of Bio-Poly P solution

Bio-Poly P (50 mg) was dissolved in 1 mL sterile Dulbecco’s Modified Eagle Medium (DMEM) (Gibco, Thermo Fisher Scientific, Waltham, United States) to achieve a 5 mg/mL concentration for subsequent application. The solution of Bio-Poly P was subjected to filtration three times through a 0.22 μm filter to ensure sterility. This sterile 5 mg/mL Bio-Poly P solution was further diluted with the DMEM to achieve the required concentrations in subsequent experiments.

### 2.4 Culture and characterization of hPDLSCs

Ethical approval (NJSH-2022NL-36) was obtained from the Ethics Committee of Nanjing Stomatological Hospital with informed consent. The sound premolars were extracted from three healthy individuals (one male and two females, Age from 14 to 20 years old) who were undergo for orthodontic procedures. Human periodontal ligament stem cells were isolated, cultured, and characterized as described in our previous paper (Qiu et al., 2021). These cells were maintained in DMEM, supplemented with 10% fetal bovine serum (FBS) (Sigma, United States) and 1% penicillin/streptomycin (HyClone, Logan, United States), at 37 °C in an atmosphere containing 5% CO_2_. Cells in passages 3 to 5 were utilized for subsequent experiments. For osteogenic differentiation, hPDLSCs (reaching 50%–60% confluence) were cultured in an osteogenic medium (OM) comprising 0.1 μM dexamethasone, 10 mM β-glycerophosphate, and 50 μM ascorbic acid-2-phosphatase (Sigma, United States). The medium was refreshed every 3 days.

The expression of specific mesenchymal cell surface markers was quantified using flow cytometry. hPDLSCs were labeled with fluorescein isothiocyanate (FITC)-conjugated monoclonal antibodies targeting CD44, CD146, CD45, and CD34 (Biolegend, China) and analyzed using FlowJo software (Tree Star, Ashland, OR).

### 2.5 Cell proliferation and viability evaluation

The effect of Bio-Poly P on the proliferation of hPDLSCs was assessed using the Cell Counting Kit-8 assay (CCK-8, Dojindo, Japan). Briefly, the hPDLSCs (P3) were seeded in 96-well plates at a density of 2000 cells/well and cultured in DMEM supplemented with 10% FBS with different concentrations of Bio-Poly P (0.25–10 mg/mL). At days 1, 3, 5, and 7 post-treatment, 10 μL of CCK-8 reagent was added to each well, which were incubated for an additional 2 h at 37 °C. The supernatant’s optical density (OD) was then measured at a wavelength of 450 nm using a SpectraMax M3 microplate reader (Molecular Devices, Sunnyvale, United States).

### 2.6 Real-time polymerase chain reaction analysis

To investigate the potential of Bio-poly P to enhance hPDLSCs osteogenic differentiation, the cells (P3) were seeded at a density of 2 × 10^5^ cells per well in 6-well plates and incubated with the osteogenic medium. On day 7, total RNA was isolated using the TRIzol reagent (Thermo Scientific, NY, United States), following the manufacturer’s protocol. Subsequently, the expression levels of osteogenesis-associated genes in hPDLSCs, including *runt-related transcription factor 2 (RUNX2), osteocalcin (OCN), osteopontin (OPN), and collagen type I alpha 1 (Col1A1),* were quantified using quantitative reverse transcription PCR (qRT-PCR). The primer sequences for *RUNX2, OCN, OPN, Col1a, and glyceraldehyde 3-phosphate dehydrogenase (GAPDH)* are listed in Table 1. Gene expression levels were calculated using the 2^^−ΔΔCT^ method (Chen et al., 2022).

**TABLE 1:** Primers used for qRT-PCR.

*Genes*	Forward primer sequence	Reverse primer sequence
*RUNX2*	CAT​CAC​TGT​CCT​TTG​GGA​GTA​G	ATG​TCA​AAG​GCT​GTC​TGT​AGG
*OCN*	ATT​GTG​GCT​CAC​CCT​CCA​TC	CCA​GCC​TCC​AGC​ACT​GTT​TA
*OPN*	CCG​AGG​TGA​TAG​TGT​GGT​TTA​TG	CTT​TCC​ATG​TGT​GAG​GTG​ATG​T
*Col1A1*	GCT​TGA​AGA​CCT​ATG​TGG​GTA​TAA	GGG​TGG​AGA​AAG​GAA​CAG​AAA

### 2.7 Alkaline phosphatase quantitative assay and alkaline phosphatase staining

hPDLSCs (2 × 10^5^ cells per well) were cultured with different culture media (according to experimental plan) in 6-well plates. On the 7th and 14th days post-treatment, the cells were lysed for 30 min on ice using a cell lysis buffer suitable for Western blotting and immunoprecipitation (IP) assays (Beyotime Biotechnology, China). The lysates were then centrifuged to separate the supernatants. The protein concentrations were determined using a BCA protein assay kit (Beyotime Biotechnology, China) following the manufacturer’s protocol. For alkaline phosphatase (ALP) activity assessment, the p-nitrophenyl phosphate (PNPP) substrate kit (Beyotime Biotechnology, China) was utilized as per the instructions provided by the company.

For the ALP staining assay, after 7 and 14 days of culture in osteogenic medium, hPDLSCs were fixed with 4% paraformaldehyde (PFA) for 15 min and subsequently washed with phosphate-buffered saline (PBS). ALP staining was conducted using the NBT/BCIP kit (Beyotime, China), and the results were observed and recorded with an Olympus camera.

### 2.8 Alizarin red staining assay

Alizarin red staining was used to detect the matrix calcium accumulation/mineralization after 18 days of culture. On day 18, hPDLSCs were washed 3 times with PBS and fixed in 4% PFA for 15 min. Then, the cells were stained with 100 mM cetylpyridinium chloride solution (Sigma, United States) for 30 min at 37 °C to visualize calcium salt deposition. When staining is finished, it was rinsed with ddH_2_O to remove residual dye and air dried for photographs to be taken.

### 2.9 Animal models and surgical procedures

A total of twenty-four *C57BL/6 mice* (four-week-old, female) were utilized in this study. The animals were housed under specific pathogen-free conditions, maintained in a 12-h light-dark cycle for 1 week. Environmental parameters were controlled, with temperatures ranging from 20 °C to 26 °C and relative humidity between 40% and 70%. All animal care procedures and experimental protocols were conducted in accordance with the guidelines approved by the Medical School of Nanjing University’s Institutional Animal Care and Use Committee. To evaluate the bone regeneration ability of Bio-Poly P, Bio-Oss^®^ (Bio-Oss bone powder, a clinically approved product and widely used for periodontal regeneration) was used as the positive control. The mice were randomly assigned into four groups (n = 6 per group): the control group (healthy mice without surgery), the bone defect group (surgery without treatment), the Bio-Poly P group (surgery followed by Bio-Poly P treatment), and the Bio-Oss group (surgery followed by Bio-Oss treatment).

**FIGURE 1:**
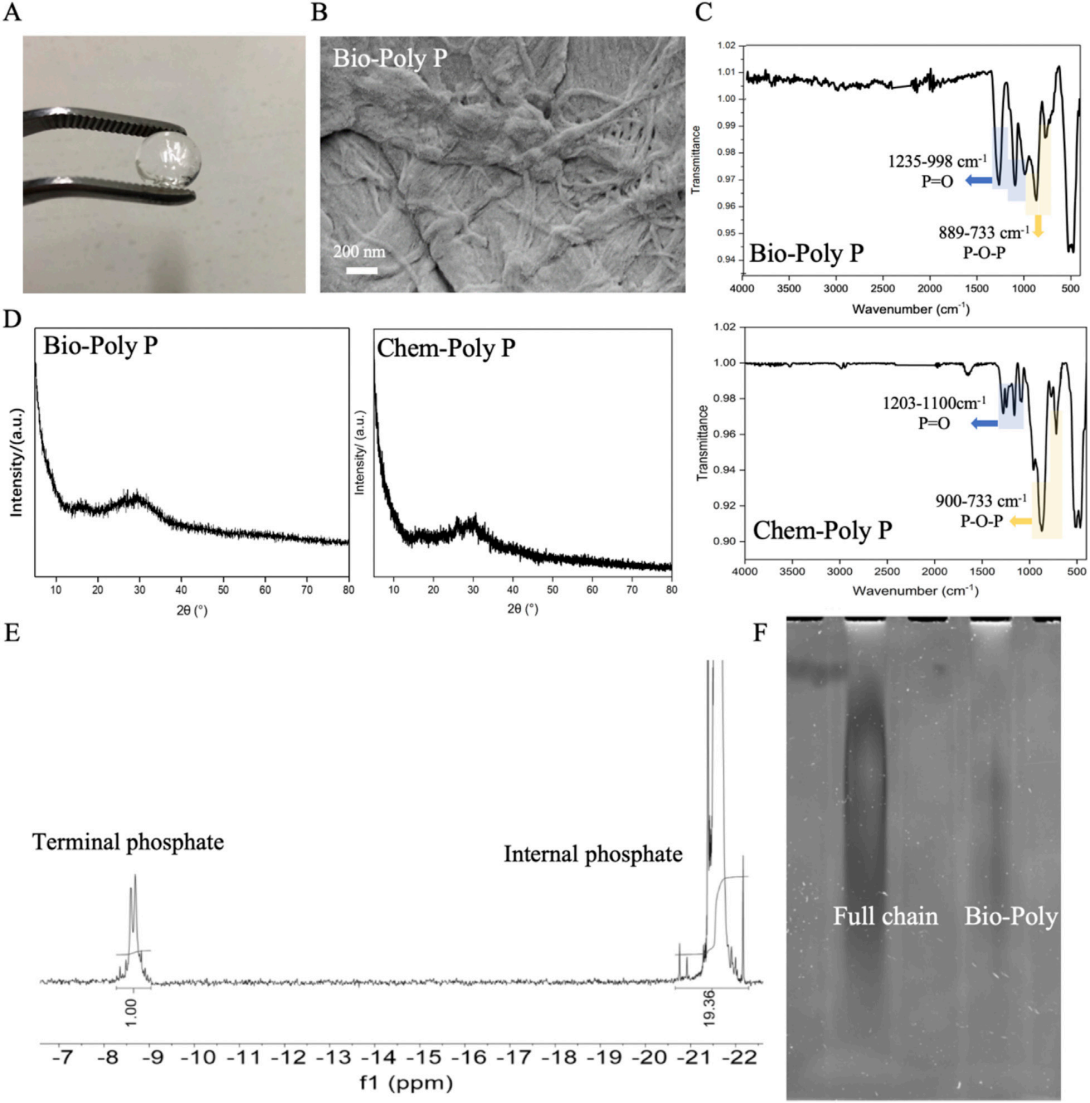
Characterization of Bio-Poly P: **(A)** Physical appearance; **(B)** SEM image; **(C)** FTIR spectra of Bio-Poly P and chemosynthetic Poly P; **(D)** PXRD patterns; **(E)** ^31^P-NMR spectra; **(F)** Urea-PAGE analysis.

To establish a mouse periodontal bone defect model, the animals were anesthetized with 7% chloral hydrate (0.4 mL/100 g) and positioned on an operating table. An incision was made along the skin of the right orofacial side in an anterior-posterior direction to expose the mandible. A defect was created using a low-speed dental drill with continuous saline irrigation until the roots of the first and second molars were exposed. The dimensions of the defect were approximately 2 mm × 1.5 mm × 1 mm. Following the creation of the defect, the wound was carefully irrigated and prepared for treatment. Bio-Poly P and Bio-Oss were sterilized using ethylene oxide before their application in surgery. The bone defect was filled with either Bio-Poly P or Bio-Oss in the treatment groups and subsequently covered with a collagen membrane. The material was crushed to powder and soaked in sterile saline before implantation. The specific procedure was shown in Figure 1. The incision was then sutured closed. Postoperative care included intramuscular administration of penicillin for 3 days. At 4 weeks post-operation, mice were first anesthetized with 3% isoflurane and subsequently sacrificed by cervical dislocation to achieve euthanasia. The entire mandibles, including the mandibular teeth, were harvested and fixed in 10% neutral buffered formalin for 48 h.

### 2.10 Micro-computed tomography scanning

The samples of mice mandibles were submerged in phosphate-buffered saline (PBS) before being scanned with a micro-computed tomography (Micro-CT) machine (Hiscan XM Micro CT, Suzhou Housefield Information Technology Co. Ltd., China). The scanning protocol included an acquisition setup of 60 kV, 134 μA, and a voxel size of 0.5 mm. CT images were conducted with a source voltage of 70 kV, a source current of 329 μA, and a slice thickness of 18 μm. Three-dimensional (3D) images were automatically reconstructed following the scan. These files were then imported into Dataviewer (Bruker, United States) for further analysis. The following parameters were assessed to evaluate new bone formation: bone volume to tissue volume ratio (BV/TV), bone surface area to tissue volume ratio (BS/TV), bone mineral density (BMD), trabecular thickness (Tb.Th), trabecular separation (Tb.Sp), and trabecular number (Tb.N). To minimize errors, the volume of tissue selected for quantification was consistent in shape and size and located in the same area across all samples.

### 2.11 HE staining

After euthanasia, the mandibles were excised from the mice to serve as specimens. Following micro-CT imaging, the mandibular samples were fixed in 10% neutral buffered formalin and then underwent standard histological processing, which included decalcification using EDTA (Servicebio, China), dehydration, embedding, and sectioning. Hematoxylin and eosin (H&E) staining (Servicebio, China) was performed to assess histological changes and osteoclastic activity within the periodontal tissues. The stained sections were scanned using a Pannoramic MIDI scanner (3DHISTECH Ltd., Budapest, Hungary) and analyzed with CaseViewer software.

### 2.12 Statistical analysis

All data are presented as mean ± standard deviation. The *in vitro* data were derived from a minimum of three independent experiments. Statistical differences were determined using a two-tailed unpaired Student’s t-test or one-way ANOVA, as appropriate. A p-value of less than 0.05 was considered statistically significant.

## 3 Results

### 3.1 Characterization of Bio-Poly P

As illustrated in Figure 1A, Bio-Poly P appeared colorless and transparent. Scanning electron microscopy (SEM) analysis (Figure 1B) revealed that Bio-Poly P exhibited a heterogeneous, irregular, and elongated braided morphology. Fourier transform infrared (FTIR) spectroscopy (Figure 1C) demonstrated characteristic absorption bands corresponding to O–P–O and P=O bonds within the 733–1,235 cm^-1^ range, indicative of phosphate groups and consistent with chemosynthetic Poly P. Powder X-ray diffraction (PXRD) patterns (Figure 1D) of both Bio-Poly P and chemosynthetic Poly P exhibited no distinct peaks, indicating an amorphous structure.

The average chain length of Bio-Poly P was assessed using phosphorus-31 (^31^P) nuclear magnetic resonance (NMR) spectroscopy and Urea-PAGE. ^31^P-NMR analysis (Figure 1E) revealed that Bio-Poly P comprised approximately 40 orthophosphate residues. Urea-PAGE results (Figure 1F) showed that the chain length distribution of Bio-Poly P corresponded to medium-chain Poly P.

### 3.2 The effect of Bio-Poly P on hPDLSCs proliferation

**FIGURE 2:**
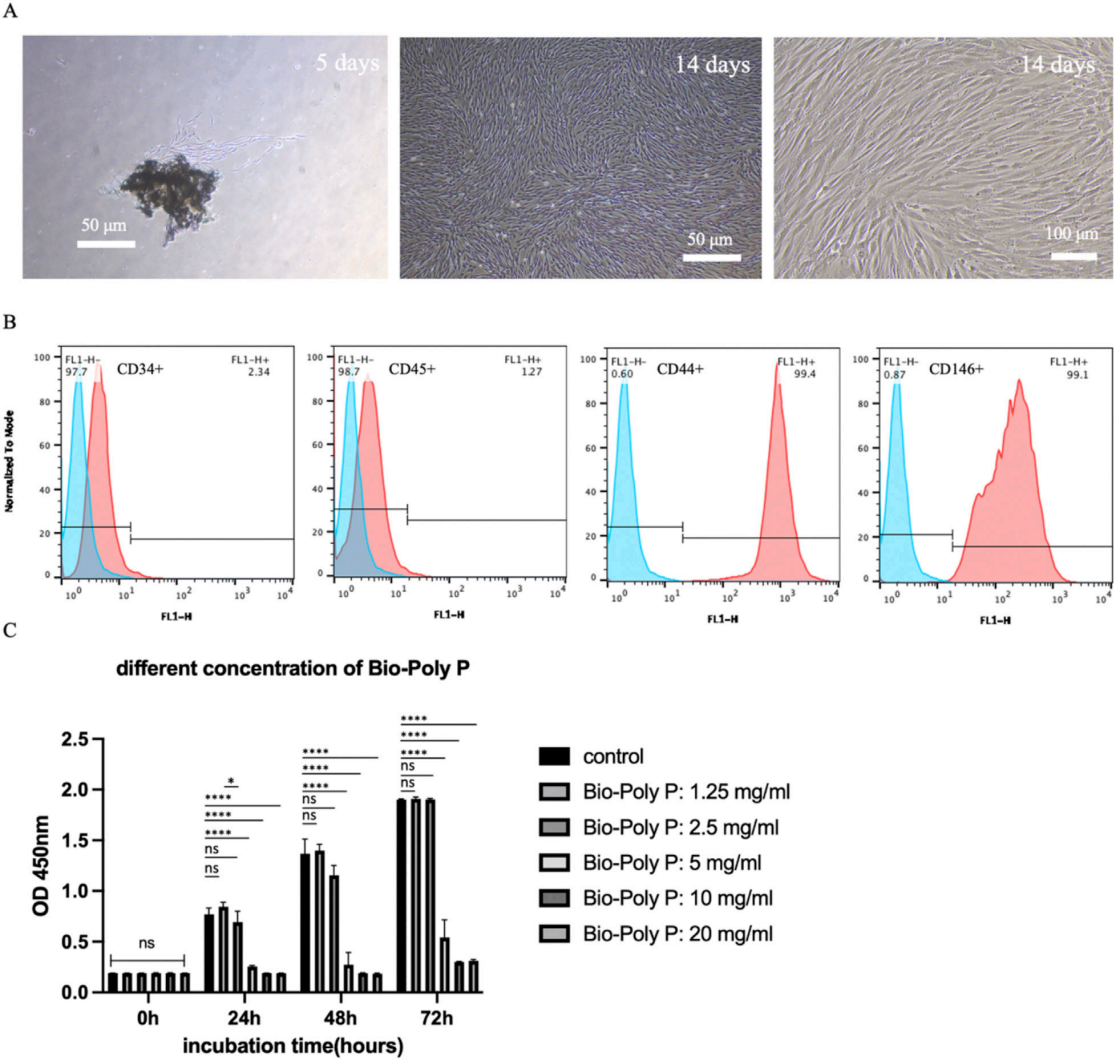
Characterization of hPDLSCs and the effect of Bio-Poly P on their proliferation: **(A)** The images of primary hPDLSCs under the microscope after culture for 5/14 days. **(B)** Analysis of cell surface antigen (negative surface antigen: CD34/45, positive: CD44/CD146) expression in hPDLSCs. **(C)** The CCK-8 result of hPDLSCs cultured with growth medium after incubation with various concentrations of Bio-Poly P. (ns = not significant, *P < 0.05, ****P < 0.0001 illustrate the significant differences between the Bio-Poly P groups and the control group).

By day 5 of primary culture, a limited number of human periodontal ligament stem cells (hPDLSCs) had migrated from the periodontal ligament tissue. These cells exhibited a characteristic spindle-shaped morphology, with abundant cytoplasm and oval-shaped nuclei, consistent with mesenchymal stem cell-like features. By day 14, the cells displayed robust growth and uniform morphology (Figure 2A). Flow cytometry analysis confirmed that over 97% of the cells expressed mesenchymal stem cell markers (e.g., positive for CD44 and CD146, and negative for CD34 and CD45) (Figure 2B).

To evaluate the cytocompatibility of Bio-Poly P, hPDLSCs were exposed to a range of concentrations, from 1.25 to 20 mg/mL. Cell Counting Kit-8 (CCK-8) assays revealed that Bio-Poly P at 1.25 and 2.5 mg/mL did not reduce the human periodontal ligament stem cells (hPDLSCs) proliferation compared to the control after 24, 48 and 72 h of culture. However, 5, 10 and 20 mg/mL groups significantly reduce the cell proliferation at all three time points compared with the control (P < 0.0001) (Figure 2C), indicating that Bio-Poly P is cytocompatible at concentrations of 1.25 and 2.5 mg/mL. Statistically significant differences were observed between 1.25 mg/mL and 2.5 mg/mL at 24 h of culture (P < 0.05), whereas no statistically significant differences were noted at both 48 h and 72 h.

### 3.3 The effect of Bio-Poly P on the osteogenic differentiation of hPDLSCs

**FIGURE 3:**
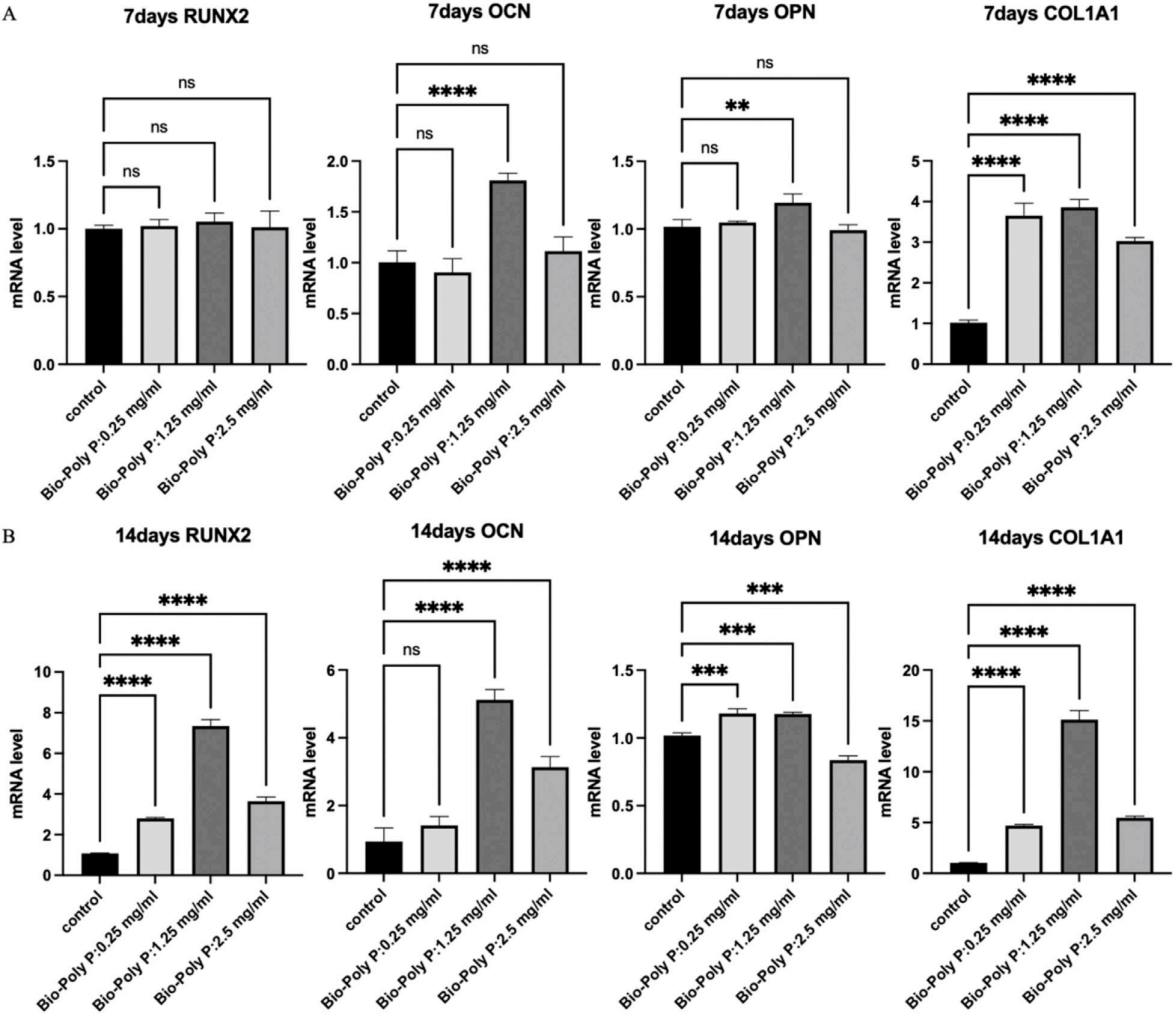
Effect of Bio-Poly P on osteogenic gene expression in hPDLSCs: **(A)** Relative expression levels on day 7; **(B)** Relative expression levels on day 14. (ns = not significant, *P < 0.05, **P < 0.01, ***P < 0.001, ****P < 0.0001 illustrate the significant differences between the Bio-Poly P groups and the control group).

Quantitative real-time PCR (qRT-PCR) was performed to evaluate the expression of osteogenic marker genes, including *RUNX2, OCN, OPN,* and *Col1A1,* on days 7 and 14 post-treatment. After 7 days, qRT-PCR results showed that there was no effect on *RUNX2* gene expression in all three groups. Only the 1.25 mg/mL Bio-Poly P group significantly upregulated the OCN (P < 0.0001) and OPN (P < 0.01) expressions. Bio-Poly P at 0.25, 1.25, and 2.5 mg/mL significantly enhanced the hPDLSCs gene expression for C*OLIA1* compared with the control (P < 0.0001) (Figure 3A). After 14 days of culture, all three groups significantly upregulated the gene expression of all osteogenic markers (*RUNX2*, *OCN*, *OPN,* and *COL1A1)* compared to the control, except the Bio-Poly P at 0.25 mg/mL group did not affect the *OCN* expression (Figure 3B).

**FIGURE 4:**
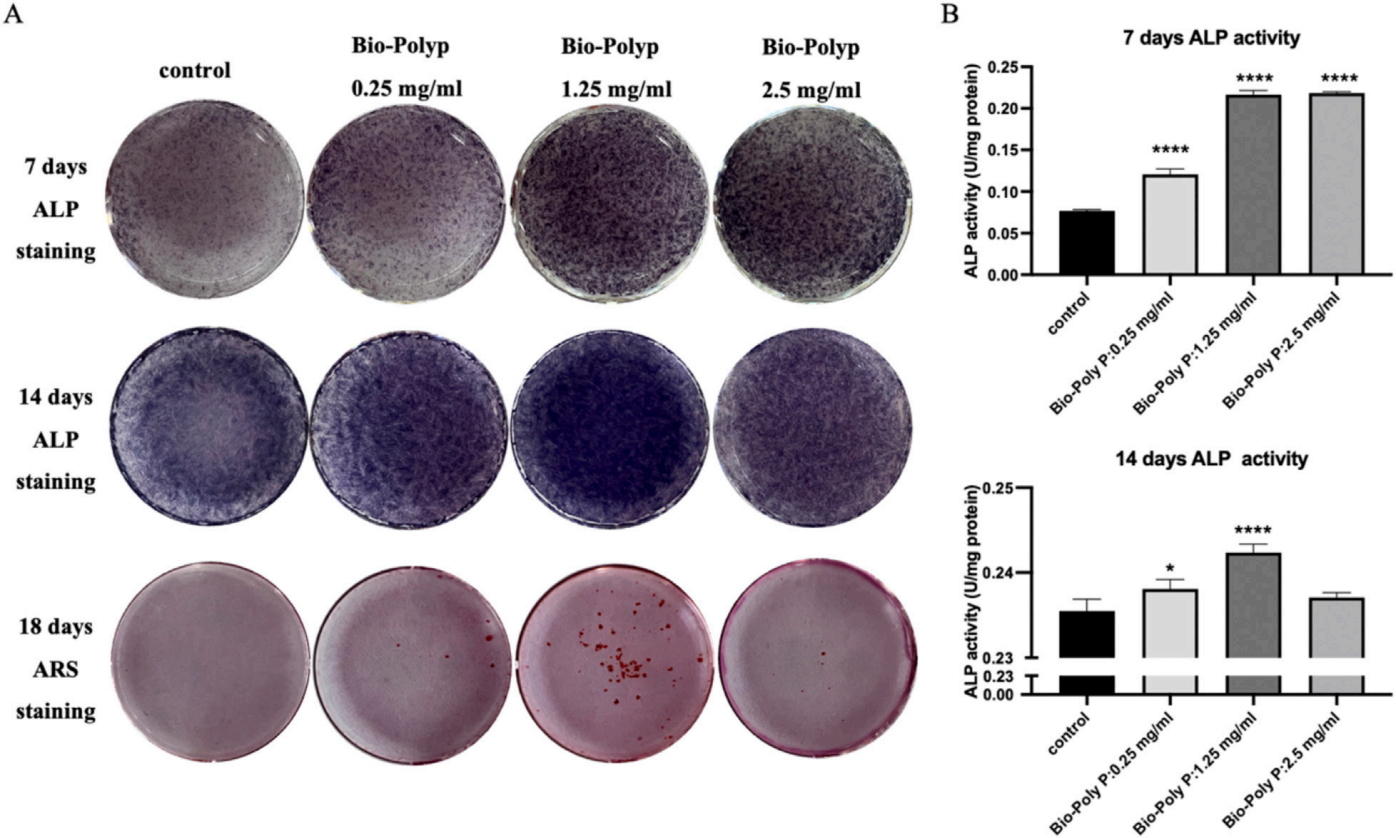
Influence of Bio-Poly P on osteogenic differentiation: **(A)** ALP staining on days 7 and 14; Alizarin Red S staining on day 18; **(B)** Quantitative analysis of ALP activity. (*P < 0.05, **P < 0.01, ***P < 0.001, ****P < 0.0001 illustrate the significant differences between the Bio-Poly P groups and the control group).

Alkaline phosphatase (ALP) staining and quantitative assays were conducted to further evaluate the effect of Bio-Poly P on the osteogenic differentiation of the hPDLSCs. After 7 and 14 days, ALP levels were substantially increased in the Bio-Poly P-treated groups compared to those in the control group, indicating enhanced osteogenic differentiation (Figures 4A,B; P < 0.05). Initially, on day 7, ALP levels and the level of osteogenetic gene expression in the groups treated with 1.25 mg/mL and 2.5 mg/mL Bio-Poly P were similar. However, by day 14, the 1.25 mg/mL concentration was significantly higher than the 2.5 mg/mL group. Additionally, Alizarin Red S staining on day 18 demonstrated increased mineralized nodule formation in the 1.25 mg/mL group, indicating enhanced extracellular calcium deposition. Alkaline phosphatase (ALP) staining and biochemical quantitative assay confirmed that Bio-Poly P 0.25 and 1.25 mg/mL groups significantly enhanced the hPDLSCs ALP activities compared with the control group at day 7 (p < 0.0001) and day 14 (p < 0.05 and p < 0.0001, respectively). However, there was no significant difference between the 2.5 mg/mL group and the control group (p > 0.05). Alizarin Red staining showed that the group with 1.25 mg/mL enhanced calcium accumulation indicated extracellular matrix mineralization compared to the untreated control and the other two test groups. There was little positive staining for Alizarin Red in the 0.25 and 2.5 mg/mL groups, and there was no staining in the control group.

### 3.4 The effect of Bio-Poly P in periodontal bone regeneration *in vivo*

**FIGURE 5:**
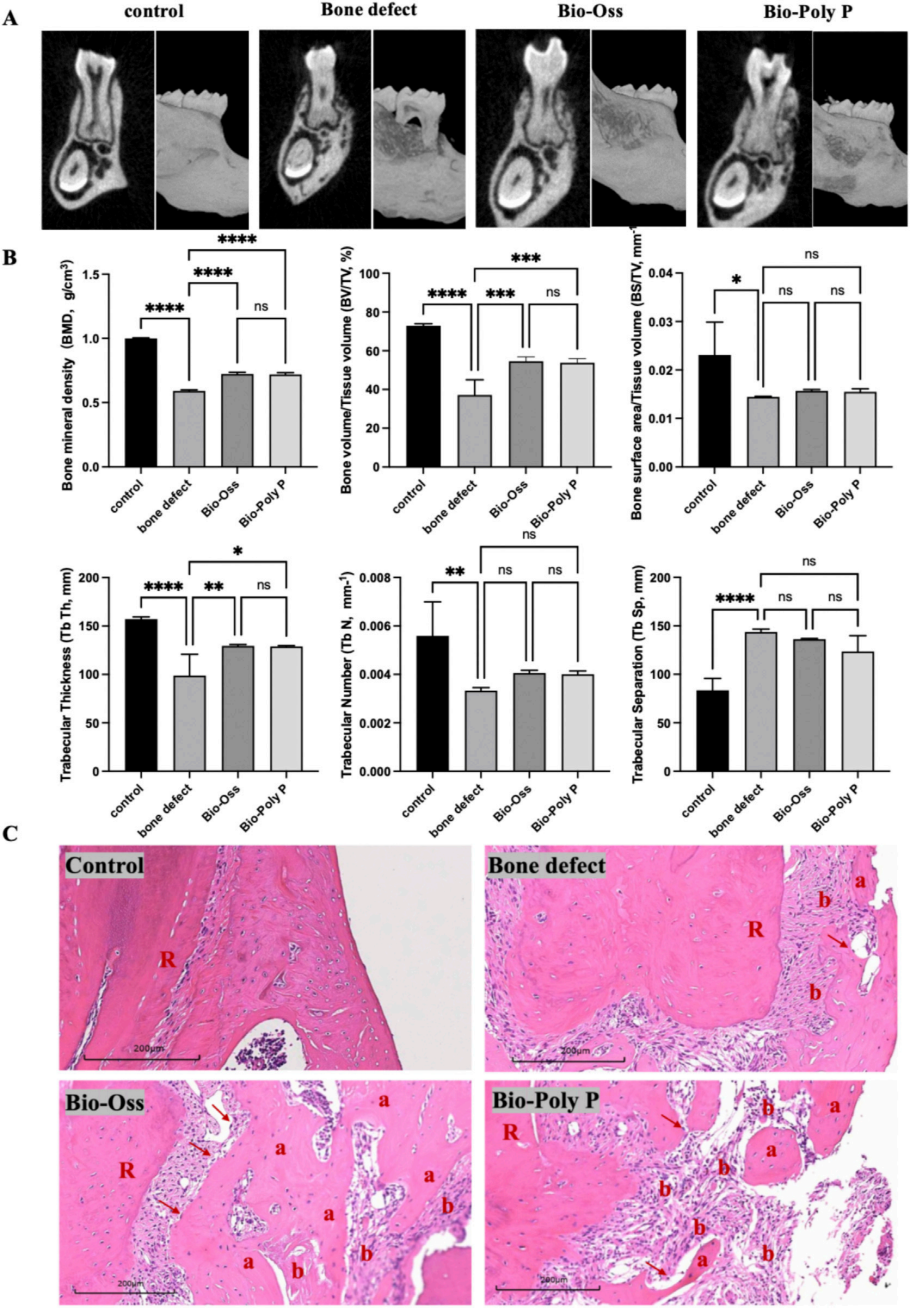
Micro-CT experiments of mice, quantification analysis and histological analysis after 4 weeks of treatment with Bio-Poly P. **(A)** Micro-CT reconstructed 3D images of the mice mandibular bone defects. **(B)** Quantification analysis of micro-CT images. **(C)** Histological images of mice mandibular bone defects. (a: new bone formation; b: colloidal tissue; R: tooth root; Red arrow: osteoblasts; ns = not significant, *P < 0.05, **P < 0.01, ***P < 0.001, ****P < 0.0001 illustrate the significant differences between the experimental groups and the control group).

A murine model of periodontal bone defect was established to evaluate the regenerative potential of Bio-Poly P. Defects were treated with either Bio-Poly P or Bio-Oss (the positive control), while a negative control group received no treatment. After 4 weeks, micro-computed tomography (Micro-CT) analysis revealed that both Bio-Poly P and Bio-Oss groups exhibited notable restoration of alveolar bone height and reduced bone resorption at the root bifurcation compared with the untreated group, which appeared to have significant alveolar bone loss (Figure 5A).

Quantitative analysis (Figure 5B) showed that the bone defect group appeared to have decreased bone mineral density (BMD), bone volume to total volume ratio (BV/TV), bone surface to total volume ratio (BS/TV), trabecular thickness (Tb.Th), and trabecular number (Tb.N), along with increased trabecular separation (Tb.Sp), confirming the successful establishment of the defect model. Bio-Poly P treatment significantly enhanced the bone mineral density (BMD), total bone volume/tissue volume ratio (BV/TV), and trabecular thickness (Tb.Sp) compared with the bone defect alone group. It is also comparable with the positive control group (Bio-Oss^®^) (P > 0.05). However, for the bone surface area/tissue volume, trabecular numbers, and trabecular separation, there were no significant differences between the Bio-Poly P, Bio-Oss groups, and the defect alone group (p > 0.05).

To further explore the periodontal bone regeneration capability of the Bio-Poly P, histological analysis of new bone formation was performed through H&E staining (Figure 5C). The bone defect areas treated with Bio-Poly P showed a substantial amount of new bone formation, a result comparable to those treated with Bio-Oss. In contrast, the control and untreated groups showed less or no new bone formation within the same time frame. A noteworthy finding was the identification of cementum-like structures within the regions treated with Bio-Poly P and Bio-Oss. These structures indicated a significant regenerative process, given that cementum regeneration was pivotal to periodontal healing and restoration. These findings suggest that Bio-Poly P has significant potential for repairing periodontal bone defects.

## 4 Discussion

Polyphosphates are widely present in biological systems and have demonstrated a positive role in promoting bone formation. However, their broader application has been limited by the complexity, high cost, and environmental concerns associated with conventional chemical synthesis methods. In this study, we utilized genetically engineered bacteria overexpressing polyphosphate kinase (PPK) to biosynthesize polyphosphates (Bio-Poly P) in a manner that is cost-effective, simple, safe, and environmentally sustainable. We further investigated the potential effects of Bio-Poly P on promoting periodontal bone regeneration.

Characterization studies revealed that Bio-Poly P shared the morphological features with the chemically synthesized polyphosphates and possesses an average chain length of approximately 40 orthophosphate residues, classifying it as a medium-chain Poly P. It is well known that medium-chain polyphosphates have the ability to enhance cell proliferation and promote osteogenic differentiation, both of which are essential for effective periodontal regeneration (Hacchou et al., 2007; Bae et al., 2016).

Biocompatibility assessments demonstrated that Bio-Poly P did not have a cytotoxic effect on the proliferation of hPDLSCs at concentrations of up to 2.5 mg/mL. Similar to our results, previous studies have reported favorable biocompatibility and safety of polyphosphates in various biomedical applications (Doi et al., 2014; Usui et al., 2010; Caswell et al., 1987). For instance, Usui et al. (Usui et al., 2010) reported that inorganic polyphosphate promotes bone regeneration without eliciting cytotoxic responses. However, higher concentrations (≥5 mg/mL) inhibited cell proliferation. This effect may result from: (1) excessive inorganic salts significantly altering the osmotic pressure of the culture medium, causing cellular dehydration or swelling that leads to stress and death (Razzaque, 2010); and (2) excessive phosphate exposure triggering apoptosis through multiple pathways, particularly via mitochondrial stress and calcium dysregulation (Di Marco et al., 2008). Therefore, concentrations ranging from 0.25 to 2.5 mg/mL were selected for subsequent *in vitro* osteogenic studies.

Our findings revealed that the Bio-Poly P significantly upregulated the expression of key osteogenic markers, including *RUNX2, OCN, OPN,* and *Col1A1*, and enhanced ALP activity and extracellular matrix mineralization in hPDLSCs. These findings are consistent with previous studies demonstrating that polyphosphates can upregulate the markers indicative of osteogenesis (Inerbaev et al., 2006).

It was suggested that the mechanisms by which polyphosphates promote osteogenesis may involve activation of fibroblast growth factor (FGF) signaling pathways, as Poly P can bind to FGF-2 and enhance its interaction with receptors (Kawazoe et al., 2008), promoting stem cell proliferation and differentiation (Zellin et al., 2000). Additionally, alkaline phosphatase-mediated hydrolysis of Poly P releases inorganic phosphate ions, which contribute to the nucleation and growth of hydroxyapatite crystals, thus facilitating mineralized tissue formation (Omelon et al., 2014). Poly P treatments have also been shown to increase extracellular calcium accumulation (Kawazoe et al., 2004; Usui et al., 2010; Banovac and Koren, 2000; Muller et al., 2011; Muller et al., 2018), and elevated intracellular calcium concentrations in osteoblasts, further supporting their role in promoting bone formation and regeneration.

*In vivo* experiments using a mouse mandibular bone defect model demonstrated that treatment with Bio-Poly P significantly improved bone repair outcomes compared to untreated bone defects. The quality of new bone, in terms of structure, mass, trabecular density, and integrity, was comparable to that observed in the Bio-Oss group, suggesting that Bio-Poly P effectively promotes osteogenesis during periodontal regeneration. These findings are consistent with previous studies that have shown the efficacy of polyphosphate-based materials in enhancing bone regeneration (Doi et al., 2014; Usui et al., 2010; Caswell et al., 1987).

Recent studies have investigated the incorporation of various metal ions into polyphosphates to develop hybrid scaffolds for bone defect repair (Li et al., 2018; Muller et al., 2017; Wang et al., 2016). These hybrid materials have exhibited superior osteogenic potential compared to polyphosphates alone, indicating a promising avenue for enhancing bone regeneration capabilities. Future research will focus on conjugating Bio-Poly P with metal ions to further improve its osteogenic effectiveness. In addition, the present study only established a simple periodontal bone defect model, whereas periodontal bone regeneration is often challenged by a chronic inflammatory microenvironment. Previous studies have reported that polyphosphates can attenuate inflammation by inhibiting macrophage M1 polarization (Li et al., 2025). Future studies should establish periodontitis models to systematically evaluate the effects of Bio-Poly P on both inflammation and bone regeneration.

## 5 Conclusion

In conclusion, our study highlights the beneficial effects of Bio-Poly P in promoting the osteogenic differentiation of hPDLSCs and enhancing the regeneration of periodontal bone defects. These findings indicated the potential of using Bio-Poly P as a feasible therapeutic option for the treatment of periodontal disease, offering new avenues for developing advanced treatment strategies to improve periodontal bone regeneration.

## Data Availability

The raw data supporting the conclusions of this article will be made available by the authors, without undue reservation.
